# The COSMO-Spain Survey: Three First Rounds of the WHO Behavioral Insights Tool

**DOI:** 10.3389/fpubh.2021.678926

**Published:** 2021-05-31

**Authors:** Carmen Rodríguez-Blázquez, María Romay-Barja, María Falcón, Alba Ayala, Maria João Forjaz

**Affiliations:** ^1^National Epidemiology Centre, Carlos III Health Institute, Madrid, Spain; ^2^Network Center for Biomedical Research in Neurodegenerative Diseases (CIBERNED), Madrid, Spain; ^3^National Centre of Tropical Medicine, Carlos III Health Institute, Madrid, Spain; ^4^Legal Medicine, Department of Sociosanitary Sciences, University of Murcia, Murcia, Spain; ^5^Department of Statistics, Universidad Carlos III de Madrid, Getafe, Spain; ^6^Health Service Research Network on Chronic Diseases (REDISSEC), Madrid, Spain

**Keywords:** COVID-19, behavioral insights, knowledge-attitude-behavior, preventive practices, self-efficacy

## Abstract

**Objective:** To describe changes in knowledge, attitudes and preventive practices (KAP), risk perception, and psychological variables of Spanish population toward the COVID-19 pandemic from July to November 2020.

**Methods:** Three samples, each of one composed by 1,000+ persons aged 18 years or older, were interviewed online in three rounds, every 2 months, from July to November 2020.

**Results:** The level of knowledge on COVID-19 was high in the three rounds, with percentages above 95% of correct answers related to ways of contagion and correct use of face masks. The most accepted measure was the mandatory use of face masks (80–86% of agreement in the three rounds, *p* = 0.001), followed by the night curfew (63% of agreement). Most participants (>80%) consistently reported using face masks, ventilating spaces, and washing or disinfecting hands. However, risk perception and self-efficacy were low. Worry about losing a loved one, the health system overload and people who do not wear face masks was high (>85% of the samples). The percentage of respondents who felt depressed due to COVID-19 increased from round 1 to round 3 (*p* = 0.044).

**Conclusions:** Spanish population has a high degree of KAP, but a relatively low risk perception and self-efficacy. These findings can help health authorities to guide containment measures and campaigns addressed to improve preventive practices.

## Introduction

The COVID-19 outbreak due to the coronavirus SARS-CoV-2 hit strongly to Spain, leading to a strict general population lockdown between March 14th and June 21st 2020, followed by several containment and preventive measures. These measures changed over time and varied between the different autonomous communities (regions) in response to their epidemiological situation. By February 2021, Spain had 3,041,454 confirmed COVID-19 cases, of which 273,717 required hospitalization and 64,217 had died (1).

In the absence of a definitive treatment and until the vaccination programs are fully implemented, the fight against the COVID-19 has been based in the behavioral changes that the preventive measures entail. Knowledge, attitudes and preventive practices (KAP) play an essential role in the control of infectious diseases such as SARS-CoV-2. Although it is a new disease, the level of knowledge about the symptoms, ways of contagion, and preventive measures seems to be good in the general population worldwide (2), with age, education level and income as main associated factors to knowledge. Positive attitudes toward preventive measures increase the willingness to adopt them and to collaborate in further prevention and control measures (3). The Theory of Planned Behavior (TPB) suggests that attitudes, perceived norms and behavioral control (perceived capacity to adhere to the norms, or self-efficacy) are the best predictors of intentions to perform a behavior (4). According to the KAP model, knowledge is the basis and attitude is the driving force of behavior change (3).

Recent studies have shown that individuals' level of perceived risk associated to the disease is key in the adherence to the preventive measures (5), and people who perceived a higher risk of getting infected were more likely to adopt protective measures (6). Psychological variables such as fear or anxiety can also have direct or indirect effects on intentions and behaviors (7). The Health Belief Model (HBM), one of the most cited theories of behavior change, include an individual's perception of susceptibility to and severity of diseases or disorders as well as the perception of benefits of and barriers to taking action and participating in prevention activities (8). Previous publications have found that more anxious or worried individuals may be more compliant with preventive measures, people who worry about a disease spontaneously keep social distance, and health concerns is associated with the adoption of preventive measures (9–11).

Behavioral insights (BI) surveys can help monitoring the KAP, risk perception, and psychological variables of the population related to COVID-19, as they are of critical importance to face the challenges posed by the pandemic, allowing to gain valuable insights into information needs, contextualize certain phenomena (e.g., acceptance of restrictions), and to target those groups needing additional attention (12). To this end, the World Health Organization (WHO) Regional Office for Europe launched in March 2020 the **CO**VID-19 **S**napshot **Mo**nitoring (COSMO) survey, together with the University of Erfurt, Germany, with the aim of providing rapid, adaptable, flexible, and regular information to authorities. Spain is one of the countries that is currently carrying out the COSMO survey every 2-months, with data currently available for 3 rounds (COSMO-Spain). The results have been shared with the Spanish health authorities and are publicly available (https://portalcne.isciii.es/cosmo-spain/).

The aim of this study was to present the (COSMO-Spain) research protocol, to describe the knowledge, attitudes, preventive practices, risk perception, and psychological variables of the Spanish population regarding COVID-19, and to analyze the evolution of those variables in three time points during the outbreak.

## Methods

### Study Protocol

Nationwide, cross-sectional panel survey on the current state of BI in Spain, specifically on knowledge, attitudes and preventive behaviors, risk perceptions, and psychological variables related to the COVID-19 pandemic. The survey was carried out by a consumer research company in three rounds, every 2 months, and each round recruited a sample of 1,000 persons matching the Spanish general population in terms of age, education, gender, and area of residence. The research company selected the sample by sending an invitation e-mail to answer an online questionnaire to the members of the panel aged 18 years or older that fit the selection criteria. People invited to answer that did not respond were replaced by others of the same stratum.

For round 1 (*n* = 1,033) data were collected between July 27th to August 4th 2020, after the first wave of the pandemic, in the midst of the “new normality,” with the mandatory use of masks implanted throughout the country and with new outbreaks in several regions (13). Some autonomous communities had specific restrictions regarding freedom of movement in some localities or areas. By August 6th, the number of COVID-19 cases was 75,146, with an accumulated incidence of 37.9 cases by 100,000 inhabitants (14).

For round 2 (*n* = 1,058), the survey was conducted between September 22nd and 25th 2020, during what has been called the beginning of the “second wave” of the coronavirus/COVID-19 pandemic. The week before, the new school year had started, with a school reopening. The cases detected during week 39 (September 21st to 27th 2020) reached to 55,877 and the accumulated incidence was 118.8 (15). The upturn in cases led to the adoption of measures to restrict activity and mobility in different cities and autonomous communities.

Round 3 (*n* = 1,018) was carried out between November 24th and 27th 2020, at the end of the “second wave” in Spain. The cases detected during that week amounted to 60,462 with a cumulative incidence of 128.6 for 14 days (16). During that week, mobility restrictions and capacity limitations in commercial establishments were maintained in different autonomous communities.

### Ethical Statement

This study was approved by the Carlos III Health Institute Ethics Committee (CEI PI 59_2020-v2). Respondents were informed about the aims of the study and participation consent was signed by accepting to fulfill the questionnaire.

### Variables

An online questionnaire was prepared to collect information on basic socio-demographic data (gender, age, education level, employment, and province), COVID-19 infection status and self-assessed health, in addition to the main study variables. The variables from the COSMO study (12) were selected and adapted taking into consideration the cultural context and the current pandemic wave (12). The survey differs slightly from one round to the other to adapt it to the epidemiological situation of the moment.

**Knowledge** on the coronavirus/COVID-19 was assessed asking the participants about the correctness of 13 statements (6 in round 1, to which 7 more were added in rounds 2 and 3) on coronavirus/COVID-19 ways of infection and symptoms, and the correct use of preventive measures. The response options were “yes,” “no,” or “do not know.”

**Attitudes** toward policies and interventions against COVID-19 were assessed by asking about the level of agreement with the adequacy of decisions taken in Spain to handle the pandemic in general, and with specific measures (mobility freedom between countries and between regions, compulsory use of face masks, opening of schools, letting the Autonomous Communities to decide the regulations). Questions were rated from 1 (strongly disagree) to 5 (strongly agree).

**Preventive behaviors** were assessed using the question “During the last 7 days, how frequently did you take the following measures to prevent infection from coronavirus/COVID-19?” in rounds 2 and 3. The scoring options were from 1 (never) to 5 (always). The listed measures were: wearing face masks according to norms and recommendations, ventilating closed spaces, using hydro alcoholic gel or disinfectants for cleaning the hands, washing my hands often with soap and water, avoiding public transportation, ensuring physical distancing (at least 2 meters), avoiding touching my eyes, nose, and mouth with unwashed hands, avoiding social/family events and disinfecting surfaces.

**Risk perception** was measured using the following questions: “what do you consider to be your own probability of getting infected with coronavirus/COVID-19?” (only in rounds 2 and 3), and the probability of getting infected in several places (public transport, meetings with family and friends, health centers, and work places), both answered from 1 (very unlikely) to 5 (very likely); and “how severe would contracting the coronavirus/COVID-19 be for you?,” answered in a scale from 1 (not severe) to 5 (very severe).

The **psychological variables** included self-efficacy, level of worry, perceived speed of propagation of the coronavirus and depression. The perceived self-efficacy (self-assessed COVID-19 self-protection and avoidance ability) was surveyed with the question “for me, avoiding an infection with coronavirus/COVID-19 in the current situation is.?,” with a response scale from 1 (very difficult) to 5 (very easy). The level of worry of the population about the coronavirus/COVID-19 outbreak in general and about specific situations was inquired. Situations included the possibility of losing a loved one (in rounds 1 and 3), the health system overload, becoming unemployed (in rounds 2 and 3), the possibility of a new lockdown, the inability to pay their bills, work/life balance problems, their own physical and mental health, going outside (in rounds 1 and 2), people that does not wear face masks (in rounds 1 and 2), closure of schools and educative centers, and family arguments for not following the rules. The question was “at the moment, how much do you worry about…?,” and the answers were rated from 1 (do not worry at all) to 5 (worry a lot). Perceived speed of propagation and depression were both questioned as “the coronavirus/COVID-19 to me feels…,” with a scale ranging from 1 (spreading slowly) and 5 (spreading fast), and from 1 (makes me feel depressed) to 5 (it does not affect my mood).

All items, originally in English, were translated by professional translators, reviewed and slightly modified by the COSMO-Spain team.

### Data Analysis

Descriptive statistics were applied to all variables. For binary and categorical response options, the percentage of participants that selected each option was computed. Mean and standard deviations were calculated for continuous variables. Data by rounds were compared using chi-squared and Mann-Whitney tests using data from the first and last available rounds.

No sampling weights were used, as the sample was representative of the Spanish population by age, sex, educative level, and area of residence.

## Results

Participants' main socio-demographic characteristics are displayed in Table 1. Mean age in round 1 was 45.7 (standard deviation, SD: 14.6; range: 18–89) years old, 45.6 (SD: 14.7; range: 18–78) in round 2 and 46.1 (SD: 14.2; range: 18–85) in round 3. Most sample participants had secondary or university studies, and half of participants was working.

**Table 1:** Socio-demographic characteristics of the samples in each round of the COSMO-Spain survey.

**Variable**	**Categories**	**Round 1**		**Round 2**		**Round 3**	
		**(*****n*** **=** **1,033)**		**(*****n*** **=** **1,058)**		**(*****n*** **=** **1,018)**	
		***n***	**%**	***n***	**%**	***n***	**%**
Sex	Women	514	49.8	533	50.4	509	50.0
	Men	519	50.2	524	49.6	509	50.0
Age groups	18–29 years	166	16.1	180	17.0	177	17.4
	30–44 years	309	29.9	310	29.3	301	29.6
	45–60 years	344	33.3	355	33.6	336	33.0
	61 years or more	214	20.7	212	20.1	204	20.0
Education level	Incomplete primary or less	17	1.6	31	3.0	31	3.0
	Primary	234	22.6	252	23.8	240	23.6
	Secondary	318	30.8	326	30.8	308	30.3
	University	464	44.9	448	42.4	439	43.1
Employment	Working	584	56.5	577	54.6	577	56.7
	Student	70	6.8	85	8.0	41	4.0
	Homemaker	78	7.6	90	8.5	32	3.1
	Retired/pensioner	154	14.9	163	15.4	186	18.3
	Long-term unemployed	91	8.8	88	8.3	100	9.8
	Unemployed or ERTE	56	5.5	54	5.1	82	8.1
Type of work^*^	With high risk of contagion	124	21.2	132	12.5	101	9.9
	With moderate risk of contagion	245	42.0	282	26.7	282	27.7
	No risk	117	20.0	69	6.5	69	6.8
	Telework	98	16.8	94	8.9	102	10.0
	Healthcare staff	–	–	–	–	23	2.3

*ERTE: Spanish Temporary Employment Regulation due to COVID-19. In Spanish, “expediente de regulación temporal de empleo.”*

* *In round 1, type of work was asked for all, whereas in rounds 2 and 3 it was only asked for those who worked*.

### Knowledge

The percentage of respondents that knew that face masks should cover nose and mouth and that hands must be washed before handling face masks increased between rounds (Table 2). However, around 10% of the participants in the three rounds incorrectly answered that face masks should be removed to cough or sneeze. Regarding the knowledge on ways of contagion, more than 90% of respondents knew that COVID-19 is spread by drops when coughing or talking in all rounds.

**Table 2:** Knowledge on coronavirus/COVID-19 in each round.

**Question**	**Round 1**	**Round 2**	**Round 3**	P^**^
If I am close contact I must isolate myself.	–	–	987 (97%)	
The flu vaccine is used to prevent covid-19.^*^	–	767 (73%)	733 (72%)	0.665
Maintaining physical distance is an effective measure.	–	995 (94%)	962 (94%)	1.000
If I am in close contact I must lead a normal life.^*^	–	–	936 (92%)	–
The recommendations of the authorities are mandatory.	–	947 (90%)	875 (86%)	0.008
COVID-19 symptoms appear as soon as you get infected.^*^	–	830 (78%)	856 (84%)	0.002
If I have symptoms, I should stay home.	–	1,000 (95%)	995 (98%)	0.003
Face masks should cover mouth and nose.	889 (86%)	1,017 (96%)	1,006 (99%)	<0.001
Hands must be washed before and after using the face mask.	785 (76%)	991 (94%)	955 (94%)	<0.001
Coronavirus/COVID-19 is spread by drops when coughing/talking.	975 (94%)	980 (93%)	976 (96%)	0.043
People who do not have fever can be contagious.	904 (87%)	803 (76%)	938 (92%)	0.001
The coronavirus is spread by physical contact with someone infected.	823 (80%)	746 (71%)	637 (63%)	<0.001
The mask must be removed to cough or sneeze.^*^	932 (90%)	894 (85%)	898 (88%)	0.171

*Frequency and percentage of correct answers*.

* *Correct answer is “no.” For questions with no asterisks, correct answer is “yes.”*

** *Chi-square test for comparing the first and last available rounds*.

### Attitudes

The perceived adequacy of measures taken to handle the pandemic decreased from 33% (round 1) to 27% (round 3) (Table 3). The measure that generated the greatest agreement was the mandatory use of face masks (80% agreement in rounds 1 and 2, and 86% on round 3), while the decision with the lowest level of agreement was the freedom of movement between countries (only 17–24% agreed with this measure).

**Table 3:** Attitudes toward preventive measures and political decisions in each round.

**Question**	**Round 1**	**Round 2**	**Round 3**	p^*^
**Agreement with the measures to reduce the spread of the**				
**coronavirus/COVID-19:**				
They have been adequate	338 (33%)	313 (30%)	273 (27%)	0.109
They have been excessive	–	167 (16%)	122 (12%)	0.337
**Agreement with the following decisions:**				
Mandatory use of face masks	822 (80%)	848 (80%)	876 (86%)	0.001
Opening of educative centers	351 (34%)	440 (42%)	532 (52%)	<0.001
Limits to the freedom of movement between provinces	393 (38%)	417 (39%)	545 (54%)	<0.001
Maintaining freedom of movement between countries	222 (21%)	256 (24%)	173 (17%)	0.317
The autonomous communities continue determining the regulations	453 (44%)	414 (39%)	429 (42%)	0.549
The closure of bars and restaurants	–	–	363 (36%)	–
Prohibition of meetings of more than 6 people	–	–	594 (58%)	–
The night curfew	–	–	639 (63%)	–

*Frequency and percentage of responses “agree” or “completely agree.”*

* *Chi-square test for comparing the first and last available rounds*.

### Preventive Behaviors

The level of adherence to recommended preventive measures was high in the three rounds. The use of face masks and avoiding public transport significantly increased from round 2 to round 3 (Table 4). Other preventive measures such as washing hands and disinfecting surfaces, significantly decreased from round 2 to round 3.

**Table 4:** Use of preventive measures in each round.

**Question**	**Round 2**	**Round 3**	p^*^
Wearing face masks according to norms and recommendations	947 (90%)	950 (93%)	0.019
Ventilating closed spaces	885 (84%)	889 (87%)	0.073
Using hydro alcoholic gel or disinfectants	895 (85%)	891 (88%)	0.064
Washing my hands often with soap and water	894 (85%)	822 (81%)	0.023
Avoiding public transport	756 (71%)	773 (76%)	0.027
Ensuring physical distance	850 (80%)	787 (77%)	0.139
Avoiding touching my eyes, nose, and mouth with unwashed hands	739 (70%)	680 (67%)	0.224
Avoiding social/family events	714 (67%)	662 (65%)	0.434
Disinfecting surfaces	584 (55%)	429 (42%)	<0.001

*Frequency and percentage of answers “almost always” and “always.” This section was not included in round 1*.

* *Chi-square test*.

### Risk Perception

Only 26% of the sample perceived their probability of contagion as high or very high, with no differences between rounds 2 and 3 (Table 5). The perceived probability of contagion when visiting crowded outdoor spaces decreased (from 52% in round 2 to 44% in round 3, *p* = 0.012), but the perception of risk when visiting crowded closed spaces increased (from 75% in round 2 to 81%, *p* = 0. 003).

**Table 5:** Risk perception in each round.

**Question**	**Round 1**	**Round 2**	**Round 3**	p^*^
**Probability of getting infected**^**a**^	–	277 (26%)	263 (26%)	1.000
**Probability of getting infected in:**^**a**^				
Public transport	679 (66%)	715 (68%)	732 (72%)	0.015
Crowded open spaces	–	549 (52%)	452 (44%)	0.012
Meetings with family and friends	524 (51%)	569 (54%)	598 (59%)	0.007
Healthcare centers	600 (58%)	536 (51%)	455 (45%)	<0.001
On-site work	495 (48%)	569 (54%)	444 (44%)	0.219
Education centers	–	512 (48%)	362 (36%)	0.001
Crowded closed spaces	–	796 (75%)	824 (81%)	0.003
**Perceived COVID-19 severity if infected**^**b**^	448 (43%)	414 (39%)	362 (36%)	0.043

a *Frequency and percentage of answers “likely” and “very likely.”*

b *Frequency and percentage of answers “severe” and “very severe.”*

* *Chi-square test comparing the first and last available rounds*.

The percentage of participants that believed they will get severe or very severe COVID-19 if infected has significantly decreased between rounds, from 43% round 1 to 36% in round 3 (*p* = 0.043).

### Psychological Variables

Most participants (64%) reported to be worried or very worried in round 1, with a non-significant decrease in rounds 2 and 3 (59%) (Table 6). The main concerns of the respondents regarding the pandemics were similar in the three rounds: the saturation of health services, losing a loved one, the people who do not use face masks, and a new general lockdown.

**Table 6:** Psychological variables in each round.

**Question**	**Round 1**	**Round 2**	**Round 3**	p^*^
**Perceived self-efficacy**^**a**^	318 (31%)	224 (21%)	244 (24%)	0.067
**Level of worry about:**^**b**^				
Coronavirus/COVID-19	662 (64%)	625 (59%)	601 (59%)	0.068
Losing a loved one	873 (85%)	–	944 (93%)	<0.001
Health system overload	838 (81%)	875 (83%)	923 (91%)	<0.001
Becoming unemployed	–	556 (53%)	509 (50%)	0.328
A new lockdown	754 (73%)	746 (71%)	682 (67%)	0.013
Inability to pay the bills	543 (53%)	632 (60%)	593 (58%)	0.090
Work and family conciliation problems	488 (47%)	601 (57%)	552 (54%)	0.024
Own physical and mental health	600 (58%)	621 (59%)	679 (67%)	0.001
Going outside	360 (35%)	451 (43%)	335 (33%)	0.578
People that does not wear face masks	827 (80%)	845 (80%)	867 (85%)	0.007
Closure of schools or educative centers	532 (52%)	608 (58%)	514 (50%)	0.518
Family arguments	337 (33%)	422 (40%)	368 (36%)	0.403
**Perceived speed of propagation**^**c**^	741 (72%)	815 (77%)	802 (79%)	0.001
**Depression**^**d**^	366 (35%)	391 (37%)	424 (42%)	0.044

a *Frequency and percentage of answers “easy” and “very easy” to the question: “Currently, being able to avoid getting infected with the coronavirus/COVID-19 is.”*

b *Frequency and percentage of answers “worried” and “very worried.”*

c *Frequency and percentage of answers “it is spreading fast.”*

d *Frequency and percentage of answers “makes me feel depressed.”*

* *Chi-square test for comparing the first and last available rounds*.

Avoiding infection with coronavirus/COVID-19 was perceived as easy or very easy only for <1 third of participants in all rounds. Most participants (72% in round 1 to 79% in round 3, *p* = 0.001) perceived the coronavirus is spreading fast. The percentage of respondents who said that COVID-19 makes them feel depressed was similar in the three rounds, with a slight increase in round 3 (42%) (*p* = 0.044).

## Discussion

The COSMO-Spain survey is the first population study focused in gathering data on knowledge, attitudes, and practices about COVID-19 in Spain. This information is of great interest for public health authorities to monitor variables that are critical to evaluate the acceptance and effectiveness of implemented measures to control transmission and to document changes over time as the pandemic progresses (12).

The study recruited three samples matched in terms of age, education, gender, and area of residence with the Spanish population. Regarding employment situation, the percentages of active and unemployed population in the three rounds of our survey are similar to those issued by the Spanish National Statistics Office: 58% of the Spanish population was working and 16% was unemployed during the third term of 2020 (17). Interestingly, the percentage of people who did telework reached 17% in the first round, decreasing to around 10% in the last rounds, reflecting the incorporation of workers to their work settings and the re-opening of schools. Although there are not official statistics on teleworking, it is estimated that around 16% of Spanish workers were working from home due to the pandemic (18).

### Knowledge

A literature review conducted at the end of July 2020, coincident with our first round, showed that most studies report a good knowledge on coronavirus/COVID-19 (2). Consistent with these studies, we found that knowledge of the population in Spain was high. Our percentage of correct answers was higher than those obtained in Malaysia in similar items during the lockdown (19). Furthermore, our results show that knowledge improved over time in most of the assessed issues, consistent with the increases in incidence and the information campaigns in Spain, focused on the correct use of face masks, awareness about asymptomatic persons and incubation period. This trend is comparable to another study conducted in the US from March to April 2020 in (20). However, two items showed a decrease of correct answers: “the recommendations of the authorities are mandatory” and “the coronavirus is spread by physical contact with someone infected.” The second one, with the lowest percentage of correct answers of all items, is of great concern and calls for information campaigns specifically addressing contacts with infected people.

### Attitudes

In general, the level of agreement with the adopted measures to control the coronavirus expansion was stable between rounds, although low, with around 30% of the interviewed that perceived them as adequate. On the contrary, only 12–16% of the participants found the measures excessive. There was a progressive acceptance of specific measures such as the mandatory use of face masks, the opening of educative centers, and the mobility restrictions, consistent with the increase in incidence and the number of cases during the second wave of the pandemic, which corresponded to round 3 of the study.

### Preventive Behaviors

Positive attitude toward preventive practices is related to the adherence and use of them (2). The use of preventive measures is high in the Spanish population, with more of 90% or people wearing face masks and more than 80% ventilating closed spaces, using hydro alcoholic gel or disinfectants and washing hands always or almost always. These results are in line with previous studies (2). It is noteworthy that in Spain, the use of face masks is compulsory in all public spaces (open or closed), with a high level of acceptance of this obligation. Disinfecting surfaces has decreased over time, probably due to the better knowledge of the COVID-19 ways of contagion, but on the contrary, avoiding the use of public transport has increased. There has been some controversy in the role public transport plays in the spread of the disease (21) and population perceives it as a setting with a high risk of contagion, as observed in our study. Measures aimed to reduce the risk of transmission, including increasing seat distance, reducing passenger density, environmental cleaning and disinfection, and use of personal hygiene protection, could contribute to rise the perception of safety of public transport during the pandemic (21, 22).

### Risk Perception

Different results were obtained according to risk perception indicators: probability of getting infected remained stable whereas perceived severity decreased. A comparative study during the lockdown identified Spain as one of the countries with the highest risk perception, using a composite score (5). The increase of perceived speed of propagation might be related to a broader geographic coverage of COVID-19 infection in Spain as time passed, as well as increased accumulated prevalence.

When analyzing risk perception in specific situations, results also varied across the surveys. Both healthcare and education centers were perceived as safer places as time elapsed. This is probably related to the strict safety measures that have been implemented in both settings in Spain, where the number of outbreaks associated to schools has been relatively low after they re-opened in September 2020 (23). The decreased risk perception in crowded open spaces (such as outdoor seating in bars and restaurants) is concerning and calls for more campaigns. The increase of risk perception in public transport might be due to the observed decrease of people teleworking in Spain and seen in the question on type of work, which led to more people using public transportation.

The mild increase of risk perception when meeting family and friends, as well as being in crowded closed spaces, might explain that, despite the high knowledge about the disease, positive attitudes and preventive behavior, the accumulated incidence in Spain had not decreased below 100 by 100,000 inhabitants during this study, maintaining the risk of transmission in a medium/high level (24). Population behavioral changes in health are also determined by the evolution of individuals risk perception of disease severity and other psychological variables.

In our study, 41–49% of participants did not perceive as (very) likely to get infected in meeting family and friends. This is congruent with the observed rate of about one third of people who reported not avoiding social/family events, despite the campaigns from Spanish health authorities. In Spain, people usually gather with family and friends, very often taken in a bar or restaurant, which might explain that only 36% agree with the closure of bars and restaurants. Besides, social contact is an import source of well-being. Taking into consideration that contact with an infected person is the major source of contagious, it is important to take measures to increase risk awareness and safety measures during social gatherings, especially in bars and restaurants, allowing people to adopt new social norms (25).

### Psychological Variables

Perceived self-efficacy remained stable and relatively low over time. This is important, as a study during the lockdown showed that it had a significant association with risk perception in Spain (5). Worry about specific situations increased in 42% of items, decreased in one item (“a new lockdown”) and remained stable in the rest of them. Consistent with the increase of worry about own physical and mental health, the rate of people reporting depressed mood also increased over time, as the restrictions tightened over time in response to the increases in incidence observed in Spain. A similar trend was observed in Argentina, comparing data gathered in March and April 2020 (26). It is important to continue monitoring the population mental health and fostering well-being.

### Study Strengths and Limitations

The main limitation of this study is that the survey was only administered online (adapted to computer or smartphone), due to logistic difficulties. This way, groups of population with problems accessing to Internet (older people, people with disabilities, or lower educative level) could be under-represented in the sample. However, the sample matched the Spanish general population in terms of age, education, gender, and area of residence. In addition, results are drawn from self-reported data. Comparison with objective data, such as direct observation of people wearing masks in a certain context, would be necessary to increase the external validity of the study.

## Conclusions

Spanish population had a high level of COVID-19 disease knowledge, positive attitudes, and preventive behavior during the pandemic. However, this seems to not have a great impact in disease transmission, which remained high. Aspects such as a low risk transmission perception in familiar situations might be delaying the change of key behaviors to the control of the transmission. Information campaigns addressed to improve prevention practices in such situations could help the population to minimize transmission risk. BI surveys such as COSMO-Spain provide relevant information about the population's knowledge, attitudes, practices, and perception that guides policy decision making. To make it effective, it is important to maintain data updated with further rounds as the COVID-19 pandemic evolves. Future aspects of interest are COVID-19 vaccine perception and epidemic fatigue.

## Data Availability Statement

The raw data supporting the conclusions of this article will be made available by the authors, without undue reservation.

## Ethics Statement

The studies involving human participants were reviewed and approved by Ethics committee of Instituto de Salud Carlos III. The participants provided their written informed consent to participate in this study.

## Author Contributions

CR-B, MJF, MR-B, and MF: conceptualization. AA-G: data curation. CR-B and AA: formal analysis. MJF: funding acquisition and project administration. CR-B: writing-original draft. All authors are writing—review and editing.

## Conflict of Interest

CR-B, MR-B, and MJF are employed in Carlos III Health Institute, the institution funding the study. The remaining authors declare that the research was conducted in the absence of any commercial or financial relationships that could be construed as a potential conflict of interest.
